# Effects of Caricaturing in Shape or Color on Familiarity Decisions for Familiar and Unfamiliar Faces

**DOI:** 10.1371/journal.pone.0149796

**Published:** 2016-02-22

**Authors:** Marlena L. Itz, Stefan R. Schweinberger, Jürgen M. Kaufmann

**Affiliations:** 1 Department of General Psychology and Cognitive Neuroscience, Friedrich Schiller University of Jena, Jena, Germany; 2 DFG Research Unit Person Perception, Friedrich Schiller University of Jena, Jena, Germany; University of Tuebingen Medical School, GERMANY

## Abstract

Recent evidence suggests that while reflectance information (including color) may be more diagnostic for familiar face recognition, shape may be more diagnostic for unfamiliar face identity processing. Moreover, event-related potential (ERP) findings suggest an earlier onset for neural processing of facial shape compared to reflectance. In the current study, we aimed to explore specifically the roles of facial shape and color in a familiarity decision task using pre-experimentally familiar (famous) and unfamiliar faces that were caricatured either in shape-only, color-only, or both (full; shape + color) by 15%, 30%, or 45%. We recorded accuracies, mean reaction times, and face-sensitive ERPs. Performance data revealed that shape caricaturing facilitated identity processing for unfamiliar faces only. In the ERP data, such effects of shape caricaturing emerged earlier than those of color caricaturing. Unsurprisingly, ERP effects were accentuated for larger levels of caricaturing. Overall, our findings corroborate the importance of shape for identity processing of unfamiliar faces and demonstrate an earlier onset of neural processing for facial shape compared to color.

## 1. Introduction

Accurate face recognition is not only relevant for social interactions on a personal level, it is also important for several occupational fields, in which identifying persons by their faces is crucial, as is the case for instance for cashiers or passport controllers (e.g. [1, 2]). While familiar face identification occurs almost effortlessly for most of us (e.g. [3]), unfamiliar face identity processing is highly error prone [4]. It has been shown repeatedly in behavioral as well as neural findings that we process unfamiliar and familiar faces in *qualitatively* different ways (for a review, see [5]). Thus, a fundamental question is whether there are facial characteristics that facilitate recognition of familiar compared to unfamiliar faces. A related and important issue for applied research (e.g. [1, 2]) is whether face identification performance can be improved in persons with poor face recognition skills, for instance by enhancing particular facial characteristics in the image.

Facial characteristics can be apportioned to two domains: shape and reflectance (see e.g. [6]). In the 2D image plane, we define shape as referring to the geometrical relationship between facial landmarks. This includes the form of individual features (e.g. eyes and nose); the second-order configuration thereof, e.g. relative metric distances between different features; as well as the overall form of a face. By contrast, reflectance refers to properties stemming from the way the skin surface and tissue underneath reflects light. This includes luminance, hue, and saturation of pixels, i.e. color-based properties. With morphing software, researchers are able to isolate and manipulate shape—by *warping*—and reflectance—by *fading—*selectively [7]. For the purpose of the current study, we use the term shape to refer to the geometrical properties of a grid that is spatially defined by the positioning of certain landmarks, whereas we use the term color to refer to the RGB values of pixels within that grid. Whereas shape, and second-order spatial configurations in particular, have long been believed to be crucial for face identification (e.g. [8]), this view has been challenged more recently (see e.g. [9]).

One intriguing possibility for examining the roles of shape and color properties is by caricaturing—a method that enhances facial distinctiveness by exaggerating idiosyncratic characteristics of an individual face, either in terms of shape, color, or both, by morphing an individual face away from an average face [10–12]. Early studies using line drawings found higher best-likeness ratings for familiar spatially caricatured compared to veridical faces [13, 14], suggesting that mental familiar face representations correspond to shape caricatures, in line with the “superportrait hypothesis” [15]. However, later studies using photorealistic stimuli found higher best-likeness ratings for slight spatial anti-caricatures, i.e. faces that had been morphed towards the average [16, 17], or for veridicals (e.g. [18]), compared to shape caricatures. Thus, mental representations of familiar faces most likely do not correspond to spatially caricatured versions.

Results from speeded recognition tasks also exhibited an inconsistent pattern of spatial caricature effects: Using line-drawn faces, Rhodes et al. [14] found faster reaction times for spatial caricatures compared to veridicals. By contrast, using photographs of both celebrity and personally familiar faces, Kaufmann and Schweinberger [18] found no differences in reaction times for spatial caricatures and veridicals. In an additional study, Lee and Perrett [17] found higher accuracies for photographic shape caricatures compared to veridicals only for very short stimulus presentation time, i.e. 33 ms. Lee and Perrett [17, 19] argued that exaggeration of idiosyncratic facial shape is only then beneficial for familiar face recognition when information is compromised somehow, either by unavailable color information, as is the case for line drawings, or when processing is hampered by time constraints such as short presentation times.

Research on color caricaturing is comparatively sparse. Lee and Perrett [19] found effects similar to those described for spatial caricatures above [17]. Specifically, accuracy advantages for color caricatures compared to veridicals were also limited to short presentation times (67 and 100 ms), albeit slightly longer than those for shape caricature advantages (33 ms). Interestingly however, Lee and Perrett [17] found higher best likeness ratings for photographs of famous faces caricatured in color compared to veridical counterparts.

Considering these findings, color information may be more diagnostic than shape information for face recognition, at least for familiar faces. For unfamiliar faces, by contrast, evidence suggests a disproportionate role for shape information. In the study by Kaufmann and Schweinberger [18], spatial caricaturing modulated ERPs for *unfamiliar* but not familiar faces. In particular, this was the case for the N250, which is associated with the processing of facial identity (e.g. [20]) and for the N170, a component associated with structural encoding (e.g. [21, 22]). These findings led the authors to hypothesize that caricaturing in shape may facilitate encoding and/or learning of initially unfamiliar faces. Follow-up face learning studies support this hypothesis, finding clear learning advantages for spatial caricatures in accuracy and/or reaction time performance and modulation of face-sensitive ERPs N170, P200, N250, and LPC [11, 23, 24]. The N170 component shows a high degree of sensitivity to faces compared to other stimulus classes [25], is typically not affected by familiarity [26–28], and is often associated with face detection and structural encoding [21, 22]. Note also that N170 is affected by facial shape [29, 30] and has been shown to be larger for shape caricatures [31, 32]. The subsequent P200 has been found to be smaller for shape caricatures [24, 31] and larger for anti-caricatures [24], and may thus be a marker of perceived shape typicality. By contrast, the N250, and the N250r (in face priming studies), have been related to the transient activation of facial representations for recognition [33]. Finally, a centro-parietal late positive component (LPC), reflects post-perceptual processing of persons rather than faces, and is typically larger for both familiar versus unfamiliar faces, and for familiar versus unfamiliar names [34, 35]. Note that both N250 and LPC are also larger for caricatured compared to veridical faces (e.g. [32]). Thus, while N250 and LPC are correlates of familiarity on the one hand, emerging evidence suggests that these components are sensitive to encoding or extraction of distinctive facial information. Moreover, spatial caricaturing effects on these ERP components were generally larger for larger levels of caricaturing (35% vs. 70%; see [32]). In a recent face learning study, strongest modulation by shape caricaturing was seen for the P200, whereas the most prominent effect of reflectance caricaturing was found for the later N250 [31]. These findings are broadly in line with the notion of earlier neural processing of facial shape than reflectance [29].

In summary, color properties may be more diagnostic for recognition of familiar faces, whereas shape may be more important for initial encoding of unfamiliar faces and earlier stages of identity-based face processing. Our first aim here was to investigate behavioral effects (in reaction times and accuracies) and underlying neural correlates of caricaturing in shape only or color only on a familiarity decision task using pre-experimentally familiar (famous) and unfamiliar faces. We also included a condition with full (shape + color) caricaturing, to test for possible supra-additive effects of shape and color (see [36]). Our second aim was to investigate the sensitivity of behavioral and ERP effects within caricature types (shape-only, color-only, or full) for the extent to which faces were caricatured (15% vs. 30% vs. 45%). Considering the previous findings mentioned above, we made the following predictions: For unfamiliar faces, we expected prominent performance benefits for shape caricatured faces, in terms of faster reaction times and higher correct rejections. For familiar faces, by contrast, we expected little or no performance differences between veridicals and caricatures. In the ERP data, we expected effects of shape caricaturing to emerge earlier than effects of color caricaturing, and were interested in whether these effects would be modulated by caricature level.

## 2. Material and Methods

### 2.1. Participants

**Fig 1:**
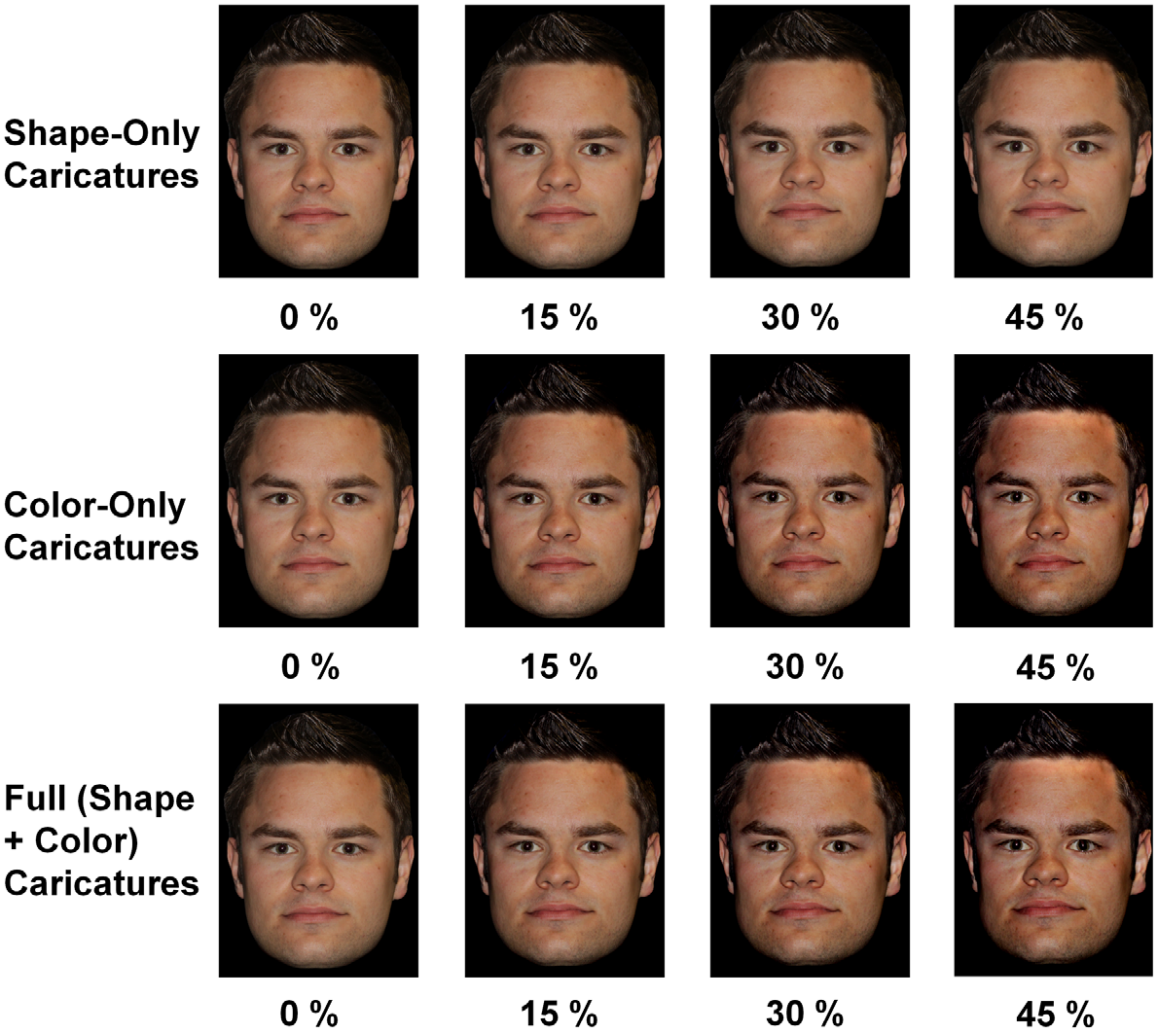
Examples of experimental stimuli. Please note that this figure depicts a single unfamiliar facial identity for the purposes of illustration only.

Data were collected from 31 participants (8 males; 2 left-handed; [37]) aged 19–31 years (*M* = 22.8, *SD* = 3.4), who reported normal or corrected-to-normal vision. Participants received either course credit or financial compensation for their participation in the study. Data from two additional participants were excluded due to insufficient EEG data quality. All participants provided written informed consent. This study, including the consent procedure, was carried out in accordance with the Declaration of Helsinki and was approved by the Ethical Commission of the Faculty of Social and Behavioural Sciences at the Friedrich Schiller University of Jena (approval number FSV 09/01). Moreover, the individual depicted in Fig 1 gave written informed consent (as outlined in PLOS consent form) to publish this figure.

### 2.2. Stimuli

Experimental stimuli comprised full-color, frontal photographs of 96 famous and 96 unfamiliar faces. Famous faces were found on the internet and unfamiliar faces were taken from the Glasgow Unfamiliar Face Database [38] and the Facial Recognition Technology (FERET) database [39, 40]. Famous and unfamiliar face sets were matched with respect to mean luminance (mean RGB value of images), *t*(190) = 1.02, *p* = .311 (*M*_*familiar*_ = 125.57, *SD*_*familiar*_ = 23.39; *M*_*unfamiliar*_ = 128.79, *SD*_*unfamiliar*_ = 20.46), and contrast (mean standard deviation of RGB values within images), *t*(190) = 0.24, *p* = .812 (*M*_*familiar*_ = 52.36, *SD*_*familiar*_ = 13.20; *M*_*unfamiliar*_ = 51.92, *SD*_*unfamiliar*_ = 11.97), prior to caricaturing. Using Adobe Photoshop^™^ (CS4, Version 11.0), we cropped faces such that only the face without the neck was visible. Using Psychomorph ([41]; http://users.aber.ac.uk/bpt/jpsychomorph/, Version 6) faces were then caricatured in either shape only, color only, or both (full; shape + color) by either 15%, 30%, or 45%. We used templates provided by Psychomorph and placed the 179 reference points of the template on standardized positions on each face (please see [42] for details on reference point placement). Caricatures were then generated such that differences with respect to shape only, color only, or both (full; shape + color) between each individual face and a gender-matched average (averages used here were those described in [43]) were exaggerated by 15%, 30%, or 45%. Note that for caricaturing of shape, color was held constant and thus unchanged, and for caricaturing of color, shape was held constant and thus unchanged; for caricaturing of both, both dimensions were changed. Images were displayed using Eprime^™^ (Version 2.0) on a black background (RGB: 0) in the center of a 16” monitor (screen resolution of 1280 x 1024 pixels). Using a chin rest, a viewing distance of 90 cm was held constant. Face stimuli size was approximately 10 cm by 7 cm for an approximate visual angle of 6° by 4.5°. Please see Fig 1 for examples of stimuli, and note that the individual depicted in Fig 1 gave written informed consent (as outlined in PLOS consent form) to publish this figure.

### 2.3. Design & Procedure

The experiment consisted of 768 trials presented in randomized order with self-paced breaks after 96 trials (8 breaks in total). For each set of famous and unfamiliar faces (96 faces each) there were 32 faces in each face type condition (shape only, color only, and full [shape + color]). Each face type condition included four caricature levels (0%, 15%, 30%, and 45%). Note that 0% caricatures were actually veridical versions of faces. Assignment of faces to each face type condition was counterbalanced across participants.

The experimental trials consisted of a white fixation cross on a black screen for 500 ms, followed by a face on a black background (presented until keypress response or for a maximum of 1500 ms), then a blank black screen for 1200 ms. Participants were instructed to indicate via keypress as accurately and quickly as possible whether each presented face was familiar or unfamiliar to them. If responses were given too slowly or not given at all within the 1500 ms time-window, “Too slow!” (“Zu langsam!” in German) appeared on the 1200 ms blank screen that followed stimulus presentation. Hand assignment (left vs. right) for familiar vs. unfamiliar answers was counterbalanced across participants. At the beginning of the experiment there were 48 practice trials with feedback ensuring that participants had understood the task. Participants were encouraged to ask any remaining questions regarding the task after the practice trials. Practice trials were not included in the data analyses.

After the experiment, a short rating procedure followed, in order to ensure participants’ familiarity with the previously seen 96 famous identities. Here faces were presented in their veridical versions coupled with the respective name and semantic information, for instance “[Name of celebrity]; Actor and film producer (Name of Film).” Participants indicated on a 6-point Likert scale (1 = very unfamiliar; 6 = very familiar) their familiarity with each of the famous identities. For each participant, only those famous identities for which at least a “3” was given were included in the analyses below (see Section 2.5 for the average number of trials per condition).

Total duration of the experiment, including EEG preparation and washing of hair afterwards was about one-and-a-half to two hours.

### 2.4. Behavioral Data

Mean accuracies and mean reaction times for correct responses were recorded and analyzed. Trials for which participants responded within the first 200 ms post-stimulus onset were excluded from the analyses.

### 2.5. Electrophysiological Recordings and Analyses

The experiment took place in an electrically shielded room. Electroencephalographic (EEG) data were recorded with sintered Ag/AgCl electrodes attached to an EasyCap^™^ electrode cap (Herrsching-Breitbrunn, Germany), arranged conforming to the extended 10/20 system at scalp positions Fz, Cz, Pz, Iz, Fp1, Fp2, F3, F4, C3, C4, P3, P4, O1, O2, F7, F8, T7, T8, P7, P8, FT9, FT10, P9, P10, PO9, PO10, F9, F10, F9′, F10′, TP9, and TP10. Cz comprised a reference, and AFz, a forehead electrode, comprised ground. Horizontal electrooculogram (EOG) signals were recorded from electrodes (F9′ and F10′) on the outer canthi of both eyes; vertical EOG signals were recorded from electrodes placed above and below the left eye. Data were amplified using SynAmps amplifiers (NeuroScan Labs, Sterling, VA) and signals were recorded with AC (0.05–100 Hz, -6 dB attenuation, 12 dB/octave), with a sampling rate of 500 Hz. Impedances were kept below 10 kΩ.

Ocular artefacts were corrected offline automatically in BESA^™^ 5.1 (Brain Electromagnetic Source Analysis, version 5.1). Epochs between -200 ms pre-stimulus onset and 1100 ms post-stimulus onset were generated, with the time interval between -200 and 0 ms serving as baseline. Trials contaminated with non-ocular artifacts (amplitude threshold of 120 μV, with a gradient criterion of 75 μV) were excluded from further analyses. Only trials with correct responses (familiar vs. unfamiliar) were analyzed. Averaged ERPs were then low-pass filtered at 20Hz (zero phase shift; 12 db/octave) and recalculated to average reference. Vertical and horizontal EOG electrodes were excluded.

ERPs were calculated relative to the 200 ms prestimulus baseline using mean amplitudes for the occipital P100 (95–135 ms), the occipitotemporal N170 (150–190 ms), P200 (210–250 ms), N250 (250–350 ms), and for a central late positive component, LPC (500–800 ms). Time intervals for P100, N170, and P200 were chosen based on distinct peaks identified in the grand mean averages across all conditions (115, 171, and 229 ms, respectively). Time-windows for N250 and LPC were chosen based on visual inspection of the means. P100 was quantified at O1/O2; N170 and P200 were quantified at PO9/PO10, P9/P10, and P7/P8; N250 was quantified at O1/O2, PO9/PO10, P9/P10, and P7/P8; and LPC was quantified at C3, Cz, and C4. In the order of caricature level (0% vs. 15% vs. 30% vs. 45%), the average numbers of trials used in the analyses were the following: for SC 25.0, 24.5, 24.3, and 24.3 (familiar faces); and 30.0, 29.0, 30.1, and 29.8 (unfamiliar faces); for CC 25.1, 25.0, 25.3, and 25.4 (familiar faces) and 29.5, 29.6, 29.5, and 29.5 (unfamiliar faces); and for FC 24.4, 24.7, 24.6, and 24.6 (familiar faces) and 29.7, 29.9, 30.1, and 30.1 (unfamiliar faces).

We used analyses of variance (ANOVA, i.e. parametric testing) to analyze our results despite violations of normality in some cases. While non-normality in parametric testing *can* lead to a Type I error (i.e. false positive results), ANOVA has been shown to be robust against violations of normality (see e.g. [44]). A larger concern for a Type I error in within-subjects ANOVA is heterogeneity of covariances. Thus, where necessary, Epsilon corrections for heterogeneity of covariances were performed throughout according to Huynh and Feldt [45]. Our analysis approach is well in line with current practice and recommendations in the field of EEG research (see e.g. [46]).

## 3. Results

Note that for pairwise comparisons (simple contrasts) of face type (i.e. SC vs. CC, SC vs. FC, & CC vs. FC), the significance level was Bonferroni-corrected to *α* = .017 [47]. Note also that polynomial trend analyses were used to assess effects of caricature level.

### 3.1. Behavioral Data

For accuracies and mean reaction times we performed 2x3x4 ANOVAs with repeated measurements on familiarity (familiar vs. unfamiliar), face type (shape-only caricatures [SC] vs. color-only caricatures [CC] vs. full (color + shape) caricatures (FC), and caricature level (0% vs. 15% vs. 30% vs. 45%). For mean reaction times, only correct responses longer than 200 ms post-stimulus onset were analyzed. For signal detection parameters (sensitivity d’ and criterion C), we performed ANOVAs analogous to those for accuracies and RTs but obviously without the factor of familiarity.

#### 3.1.1. Accuracies

Accuracies were highest for 30% and 45% unfamiliar shape caricatures. The ANOVA yielded a main effect of familiarity, *F*(1,30) = 25.55, *p* < .001, η_p_^2^ = .460, which interacted with face type and caricature level, *F*(6,180) = 3.08, *p* = .014, η_p_^2^ = .093, ε_HF_ = .759. Separate ANOVAs for familiar and unfamiliar faces were thus conducted. For familiar faces there was just a trend for the interaction of face type by caricature level, *F*(6,180) = 2.07, *p* = .063, η_p_^2^ = .065, ε_HF_ = .941, whereas for unfamiliar faces this interaction was significant, *F*(6,180) = 2.34, *p* = .047, η_p_^2^ = .072, ε_HF_ = .796. Separate analyses for each unfamiliar face type revealed a main effect of caricature level for shape caricatures only, *F*(3,90) = 3.04, *p* = .039, η_p_^2^ = .092, ε_HF_ = .898, due to a cubic trend, *F*(1,30) = 6.78, *p* = .014, η_p_^2^ = .184 (Table 1).

**Table 1:** Behavioral Data.

		Face Type		
	Caricature Level (CL)	SC	CC	FC
ACC (familiar)	0%	.915 (.016)	.910 (.017)	.887 (.018)
	15%	.915 (.014)	.912 (.015)	.902 (.016)
	30%	.894 (.018)	.916 (.014)	.911 (.016)
	45%	.901 (.016)	.916 (.017)	.913 (.013)
	Averaged across CL	.906 (.015)	.913 (.015)	.903 (.014)
ACC (unfamiliar)	0%	.977 (.007)	.973 (.006)	.977 (.006)
	15%	.964 (.009)	.970 (.008)	.986 (.005)
	30%	.984 (.005)	.964 (.008)	.982 (.005)
	45%	.983 (.004)	.969 (.008)	.978 (.007)
	Averaged across CL	.977 (.005)	.969 (.006)	.981 (.004)
RTs (familiar)	0%	656 (18)	656 (17)	659 (18)
	15%	648 (17)	645 (17)	653 (17)
	30%	660 (17)	643 (16)	655 (18)
	45%	651 (15)	652 (17)	659 (18)
	Averaged across CL	654 (16)	649 (16)	656 (17)
RTs (unfamiliar)	0%	624 (19)	630 (19)	636 (20)
	15%	621 (16)	629 (18)	626 (19)
	30%	625 (20)	639 (20)	635 (21)
	45%	622 (19)	655 (20)	639 (20)
	Averaged across CL	623 (18)	638 (19)	634 (20)
*d’*	0%	3.41 (0.11)	3.34 (0.12)	3.23 (0.10)
	15%	3.27 (0.11)	3.27 (0.11)	3.37 (0.10)
	30%	3.31 (0.11)	3.27 (0.10)	3.40 (0.09)
	45%	3.36 (0.10)	3.36 (0.12)	3.35 (0.09)
	Averaged across CL	3.34 (0.09)	3.31 (0.10)	3.34 (0.09)
*C*	0%	0.19 (0.05)	0.18 (0.05)	0.27 (0.05)
	15%	0.16 (0.04)	0.17 (0.05)	0.26 (0.04)
	30%	0.28 (0.04)	0.15 (0.05)	0.23 (0.05)
	45%	0.26 (0.05)	0.16 (0.05)	0.22 (0.04)
	Averaged across CL	0.22 (0.04)	0.16 (0.04)	0.25 (0.04)

Note: Mean accuracies (proportion correct) and reaction times (RTs; in milliseconds) for face types, shape-only caricatures (SC), color-only caricatures (CC), and full (shape + color; FC) caricatures, and caricature levels within each face type (0%, 15%, 30%, and 45%); *d’* and *C* are listed also. Values in parentheses represent standard errors of the means (SEMs).

#### 3.1.2. Mean reaction times

Mean reaction times were fastest for unfamiliar shape caricatures. The ANOVA revealed main effects of familiarity, *F*(1,30) = 4.28, *p* = .047, η_p_^2^ = .125, and face type, *F*(2,60) = 4.07, *p* = .022. η_p_^2^ = .119, and an interaction between those two factors, *F*(2,60) = 5.84, *p* = .005, η_p_^2^ = .163. Separate analyses for both familiar and unfamiliar faces yielded a main effect of face type for unfamiliar faces only, *F*(2,60) = 9.97, *p* < .001, η_p_^2^ = .249. Simple contrasts revealed faster reaction times for unfamiliar SC compared to both CC, *F*(1,30) = 21.74, *p* < .001, η_p_^2^ = .420, and FC, *F*(1,30) = 9.73, *p* = .004, η_p_^2^ = .245, with no difference between CC and FC, *F*(1,30) = 1.18, *p* = .287, η_p_^2^ = .038 (Table 1).

Note that in the overall ANOVA there was also a main effect of caricature level, *F*(3,90) = 2.88, *p* = .041, η_p_^2^ = .087, due to a quadratic tend, *F*(1,30) = 4.60, *p* = .040, η_p_^2^ = .133.

#### 3.1.3. Signal detection measurements (d’ and C)

Participants responded somewhat more conservatively to full (color + shape) caricatures.

Analyses for signal detection measurements yielded no significant main effects or trends for d´, but revealed a main effect of face type for criterion C, *F*(2,60) = 3.98, *p* = .030, η_p_^2^ = .117, ε_HF_ = .860. Simple contrasts yielded higher criterion C for FC compared to CC, *F*(1,30) = 14.27, *p* = .001, η_p_^2^ = .322, with no difference between SC and FC, *F*(1,30) = 0.55, *p* = .464, η_p_^2^ = .018, and only a numeric difference between SC and CC, *F*(1,30) = 3.04, *p* = .091, η_p_^2^ = .092 (see Table 1). Moreover, there was a trend for the interaction of face type by caricature level for criterion C, *F*(6,180) = 2.25, *p* = .066, η_p_^2^ = .070, ε_HF_ = .681.

### 3.2. Electrophysiological Results

For the ERP data, we performed analyses analogous to the behavioral data. For P100, N170, P200, and N250, the additional factors of electrode site and hemisphere were included. For LPC, the additional factor of laterality was included. For readability and stringency we report only those results pertaining to experimental factors of familiarity, face, and caricature level. Thus, main effects and interactions involving solely site and/or hemisphere are not reported.

#### 3.2.1. P100

**Fig 2:**
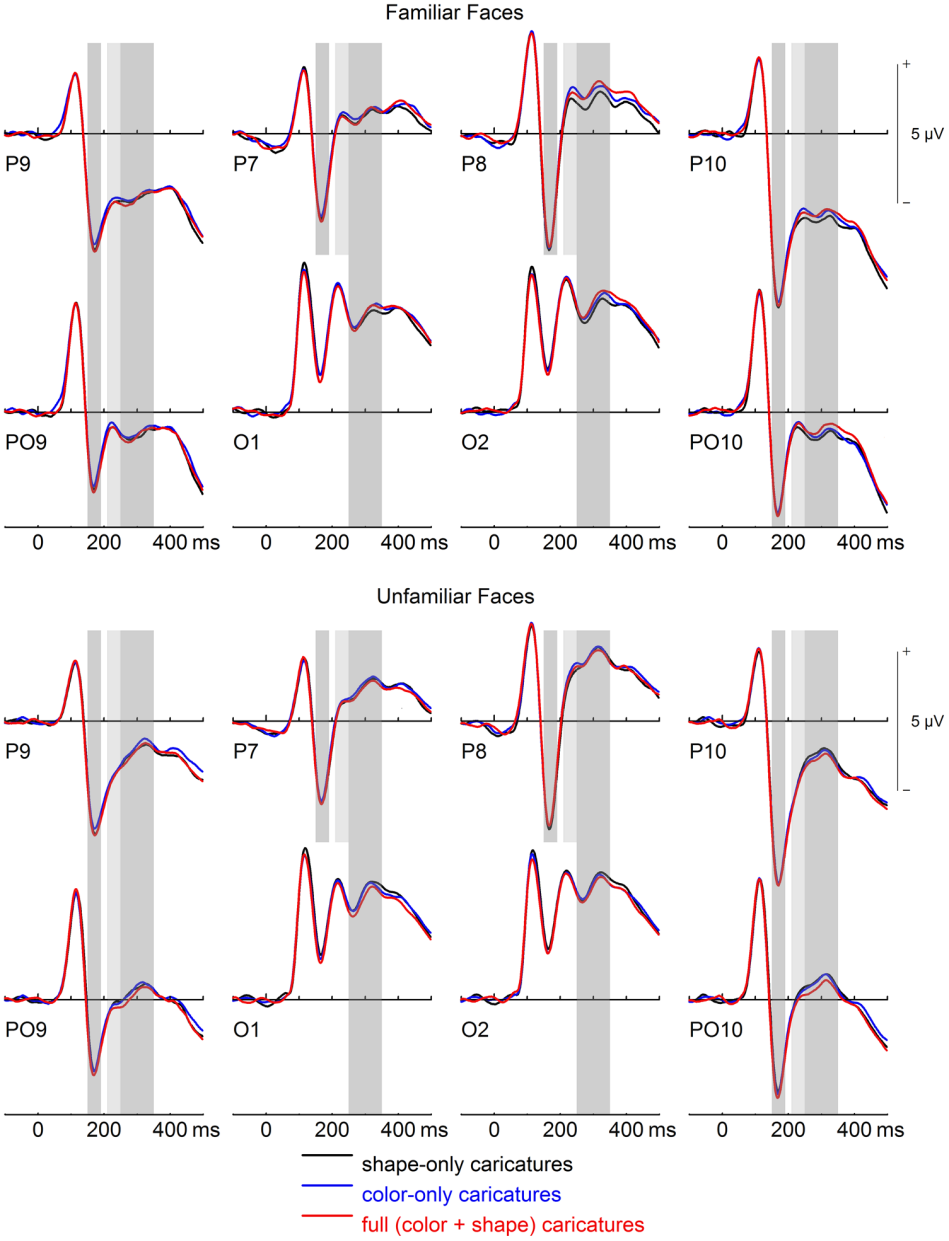
ERPs P100, N170, P200, and N250 for familiar and unfamiliar faces. Gray-shaded areas denote time-windows of interest for N170 (150–190 ms), P200 (210–250 ms), and N250 (250–350 ms). Note smallest P200 for shape-only caricatures at P8.

For P100, a main effect of face type was found, *F*(2,60) = 5.15, *p* = .009, η_p_^2^ = .146. Simple contrasts revealed slightly larger amplitudes for SC (mean = 4.74 μV) than FC (mean = 4.47 μV), *F*(1,30) = 9.96, *p* = .004, η_p_^2^ = .249, and numerically larger amplitudes for SC than CC (Mean μV = 4.55), *F*(1,30) = 4.45, *p* = .043, η_p_^2^ = .129 (see Fig 2). There was no difference between CC and FC, *F*(1,30) = 0.84, *p* = .368, η_p_^2^ = .027. There were also trends for main effects of familiarity, *F*(1,30) = 3.41, *p* = .075, η_p_^2^ = .102, and caricature level, *F*(3,90) = 2.21, *p* = .092, η_p_^2^ = .069, and for the interaction between those two factors, *F*(3,90) = 2.48, *p* = .077, η_p_^2^ = .076, ε_HF_ = .845.

#### 3.2.2. N170

**Fig 3:**
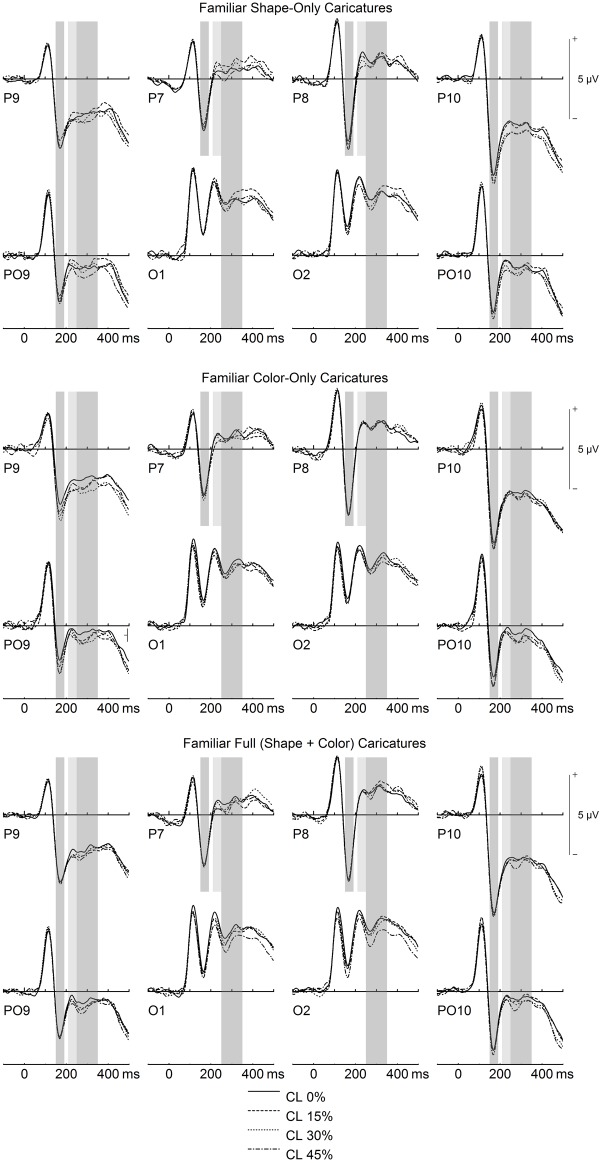
Early ERP Caricature level effects for familiar faces. Gray-shaded areas denote time-windows of interest for N170 (150–190 ms), P200 (210–250 ms), and N250 (250–350 ms).

**Fig 4:**
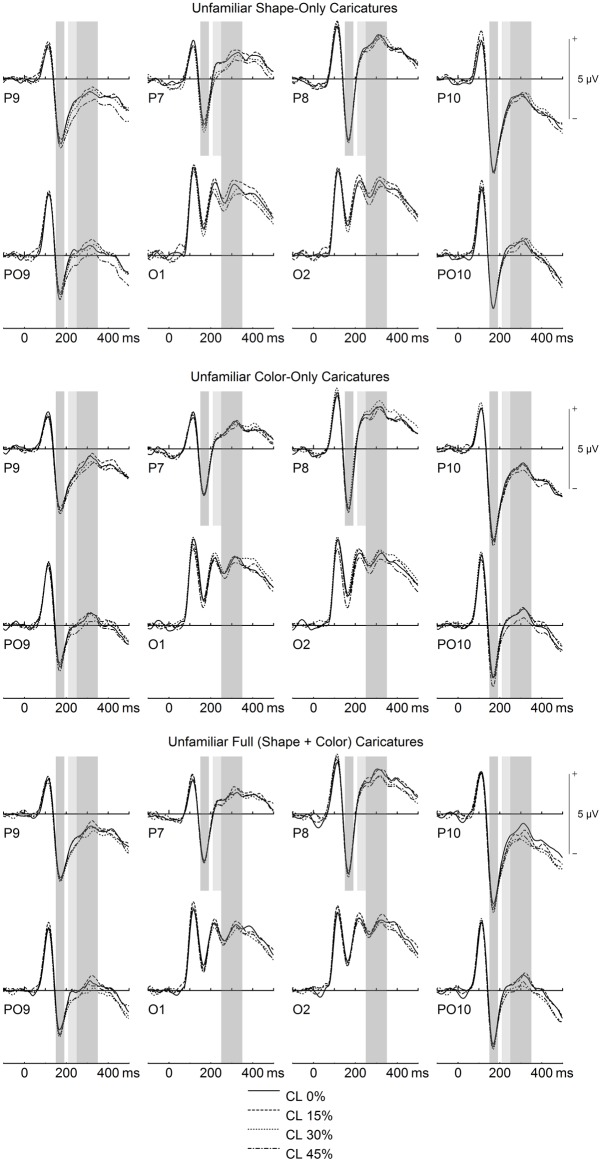
Early ERP caricature level effects for unfamiliar faces. Gray-shaded areas denote time-windows of interest for N170 (150–190 ms), P200 (210–250 ms), and N250 (250–350 ms). Note decreasing P200 with increasing caricature level for shape-only and full (shape + color) caricatures.

N170 amplitudes were larger for familiar compared to unfamiliar faces overall, and for increasing caricature level (see Figs 3 & 4).

The analysis for N170 yielded a main effect of familiarity, *F*(1,30) = 18.28, *p* < .001, η_p_^2^ = .379, due to larger amplitudes for familiar compared to unfamiliar faces. The main effect of caricature level, *F*(3,90) = 5.33, *p* = .004, η_p_^2^ = .151, ε_HF_ = .817, interacted with site, *F*(6,180) = 3.63, *p* = .007, η_p_^2^ = .108, ε_HF_ = .682. Also, we found an interaction between site and face type, *F*(4,120) = 3.24, *p* = .024, η_p_^2^ = .098, ε_HF_ = .772.

Separate ANOVAs for each site were performed to disentangle the aforementioned interactions. No main effects of face type were found, *F*s(2,60) < 1.44, *p*s > .099, η_p_^2^s < .075. Main effects of caricature level were found for sites P9/P10 and PO9/PO10, *F*s(3,90) > 5.83, *p*s < .002, η_p_^2^s > .162 (ε_HF_ = .836 for PO9/PO10), due to linear trends, *F*s(1,30) > 15.37, *p*s < .001, η_p_^2^s > .354 (see Figs 3 & 4). No main effect of caricature level was found for P7/P8, *F*(3,90) = 0.72, *p* = .530, η_p_^2^ = .023, ε_HF_ = .906.

#### 3.2.3. P200

P200 was smaller for familiar compared to unfamiliar faces overall. In terms of face type, P200 was smallest for shape caricatures, although this was restricted to electrode P8 (see Fig 2). Moreover, effects of caricature level were strongest for unfamiliar shape caricatures (see Fig 4).

The ANOVA for P200 yielded main effects of familiarity, *F*(1,30) = 29.96, *p* < .001, η_p_^2^ = .500, and caricature level, *F*(3,90) = 9.92, *p* < .001, η_p_^2^ = .249. Familiarity was further qualified by interactions, site x familiarity, *F*(2,60) = 27.33, *p* < .001, η_p_^2^ = .477, ε_HF_ = .797, and hemisphere x familiarity, *F*(1,30) = 4.84, *p* = .036, η_p_^2^ = .139. Caricature level was further qualified by the interaction, site x caricature level, *F*(6,180) = 3.03, *p* = .023, η_p_^2^ = .092, ε_HF_ = .620. These aforementioned effects were then qualified further by the four-way interaction of site x hemisphere x familiarity x caricature level, *F*(6,180) = 2.37, *p* = .037, η_p_^2^ = .073, ε_HF_ = .091: We thus conducted analyses for each separate electrode to disentangle this four-way interaction. Main effects of familiarity were found for all sites except for P7, *F*s(1,30) > 5.21, *p*s < .029, η_p_^2^s > .147, due to smaller P200 for familiar compared to unfamiliar faces. Moreover, at all sites except for P8 there were main effects of caricature level, *F*s(3,90) > 4.35, *p*s < .010, η_p_^2^s > .126 (ε_HF_ = .825 for P7), due to linear trends, *F*s(1,30) > 10.26, *p*s < .004, η_p_^2^s > .254. Finally, at P9 there was a trend for the interaction of familiarity by caricature level, *F*(3,90) = 2.49, *p* = .065, η_p_^2^ = .077.

With respect to *face type*, the three-way interaction of site x hemisphere x face type, *F*(4,120) = 3.21, *p* = .015, η_p_^2^ = .097, was significant. Analyses for each separate electrode yielded a main effect of face type at P8 only, *F*(2,60) = 5.55, *p* = .006, η_p_^2^ = .156, due to smallest P200 for shape caricatures (see Fig 2): Simple contrasts yielded smaller P200 for SC compared to CC, *F*(1,30) = 12.29, *p* = .001, η_p_^2^ = .291, with no differences for FC versus CC, *F*(1,30) = 3.30, *p* = .079, η_p_^2^ = .099, and SC versus FC, *F*(1,30) = 2.31, *p* = .139, η_p_^2^ = .072.

Moreover, the four-way interaction, hemisphere x familiarity x face type x caricature level, *F*(6,180) = 2.53, *p* = .023, η_p_^2^ = .078, was significant: Separate ANOVAs for each familiarity level over each hemisphere were thus performed. For familiar faces over the left hemisphere (LH), there was a trend for the main effect of caricature level, *F*(3,90) = 2.47, *p* = .067, η_p_^2^ = .076, and a significant interaction between face type and caricature level, *F*(6,180) = 2.60, *p* = .019, η_p_^2^ = .080. Separate analyses were thus conducted for each familiar face type over the LH. For familiar faces over the LH, we found the following: A main effect of caricature level for SC, *F*(1,30) = 3.32, *p* = .023, η_p_^2^ = .100, due to a quadratic trend, *F*(1,30) = 5.64, *p* = .024, η_p_^2^ = .158 (Fig 3); a trend for the main effect of caricature level for CC, *F*(3,90) = 2.42, *p* = .072, η_p_^2^ = .075; and no effect of caricature level for FC, *F*(3,90) = 1.96, *p* = .127, η_p_^2^ = .061. For familiar faces over the right hemisphere (RH), there was a main effect of caricature level, *F*(3,90) = 2.75, *p* = .047, η_p_^2^ = .084, due to a linear trend, *F*(1,30) = 8.98, *p* = .005, η_p_^2^ = .230, and no effects of face type. Over the LH for unfamiliar faces there was a main effect of caricature level, *F*(3,90) = 8.78, *p* < .000, η_p_^2^ = .226, ε_HF_ = .856, which interacted with face type, *F*(6,180) = 3.25, *p* = .005, η_p_^2^ = .098: Main effects of caricature level were found for unfamiliar SC, *F*(3,90) = 12.74, *p* < .001, η_p_^2^ = .298, and FC, *F*(3,90) = 3.35, *p* = .022, η_p_^2^ = .100, due to linear trends (*F*[1,30] = 38.48, *p* < .001 η_p_^2^ = .562 for SC, and *F*[1,30] = 12.56, *p* = .001, η_p_^2^ = .295 for FC; Fig 4). Finally, over the RH for unfamiliar faces there was a main effect of caricature level, *F*(3,90) = 3.43, *p* = .020, η_p_^2^ = .103, due to a linear trend, *F*(1,30) = 10.48, *p* = .003, η_p_^2^ = .259, with no effects of face type.

Note that in the overall ANOVA there were also trends for the interactions, site x face type, *F*(4,120) = 2.11, *p* = .096, η_p_^2^ = .066, ε_HF_ = .837, and face type x caricature level, *F*(6,180) = 1.98, *p* = .071, η_p_^2^ = .062.

#### 3.2.4. N250

For the N250 time-window, amplitudes were larger for familiar compared to unfamiliar faces overall. Moreover, effects of caricature level were strongest for shape and color caricatures over the left hemisphere, and for full (shape + color) caricatures over the right hemisphere (see Figs 3 & 4).

The ANOVA yielded a prominent main effect of familiarity, *F*(1,30) = 73.35, *p* < .001, η_p_^2^ = .710, which was further qualified by interactions, site x familiarity, *F*(3,90) = 38.87, *p* < .001, η_p_^2^ = .564, ε_HF_ = .553, and site x hemisphere x familiarity, *F*(3,90) = 5.39, *p* = .006, η_p_^2^ = .152, ε_HF_ = .695. Separate ANOVAs for each electrode yielded main effects of familiarity at electrodes, P7, P8, P9, P10, PO9, and PO10, *F*s(1,30) > 15.08, *p*s < .001, η_p_^2^s > .334, due to larger N250 for familiar compared to unfamiliar faces. At both O1 and O2, the main effects of familiarity were just trends (*F*[1,30] = 3.66, *p* = .065, η_p_^2^ = .109 for O1, and *F*[1,30] = 3.21, *p* = .083, η_p_^2^ = .097 for O2).

There was also a main effect of caricature level, *F*(3,90) = 10.74, *p* < .001, η_p_^2^ = .264, which was further qualified by the interactions site x caricature level, *F*(9,270) = 2.75, *p* = .008, η_p_^2^ = .084, ε_HF_ = .839, and hemisphere x face type x caricature level, *F*(6,180) = 2.57, *p* = .021, η_p_^2^ = .079. The two-way interaction between site and caricature level was followed up with separate analyses for each site: All sites yielded main effects of caricature level, *F*s(3,90) > 4.84, *p*s < .005, η_p_^2^s > .138, due to linear trends, *F*s(1,30) > 11.05, *p*s < .003, η_p_^2^s > .268 (Figs 3 & 4). Note that the quadratic trend was also significant for site O1/O2, *F*(1,30) = 5.01, *p* = .033, η_p_^2^ = .143.

To disentangle the latter three-way of hemisphere x face type x caricature level, separate analyses were performed for each face type over both hemispheres. Over the left hemisphere, there were main effects of caricature level for SC, *F*(3,90) = 10.81, *p* < .001, η_p_^2^ = .265, and CC, *F*(3,90) = 3.42, *p* = .021, η_p_^2^ = .102, due to linear trends (*F*[1,30] = 24.06, *p* < .001, η_p_^2^ = .445 for SC, and *F*[1,30] = 11.01, *p* = .002, η_p_^2^ = .268 for CC; Figs 3 & 4). Note that for SC, the quadratic trend was also significant, *F*(1,30) = 8.39, *p* = .007, η_p_^2^ = .219, and there was a trend for a cubic trend, *F*(1,30) = 3.26, *p* = .081, η_p_^2^ = .098. Moreover, over the left hemisphere, there was a trend for caricature level for FC, *F*(3,90) = 2.29, *p* = .091, η_p_^2^ = .071, ε_HF_ = .897. Over the right hemisphere, there was a significant main effect of caricature level for FC only, *F*(3,90) = 5.53, *p* = .002, η_p_^2^ = .156, due to a linear trend, *F*(1,30) = 11.07, *p* = .002, η_p_^2^ = .270 (Figs 3 & 4). Over the right hemisphere, there were also trends for main effects of caricature level for SC, *F*(3,90) = 2.36, *p* = .077, η_p_^2^ = .073, and CC, *F*(3,90) = 2.53, *p* = .074, η_p_^2^ = .078, ε_HF_ = .839.

#### 3.2.5. LPC

**Fig 5:**
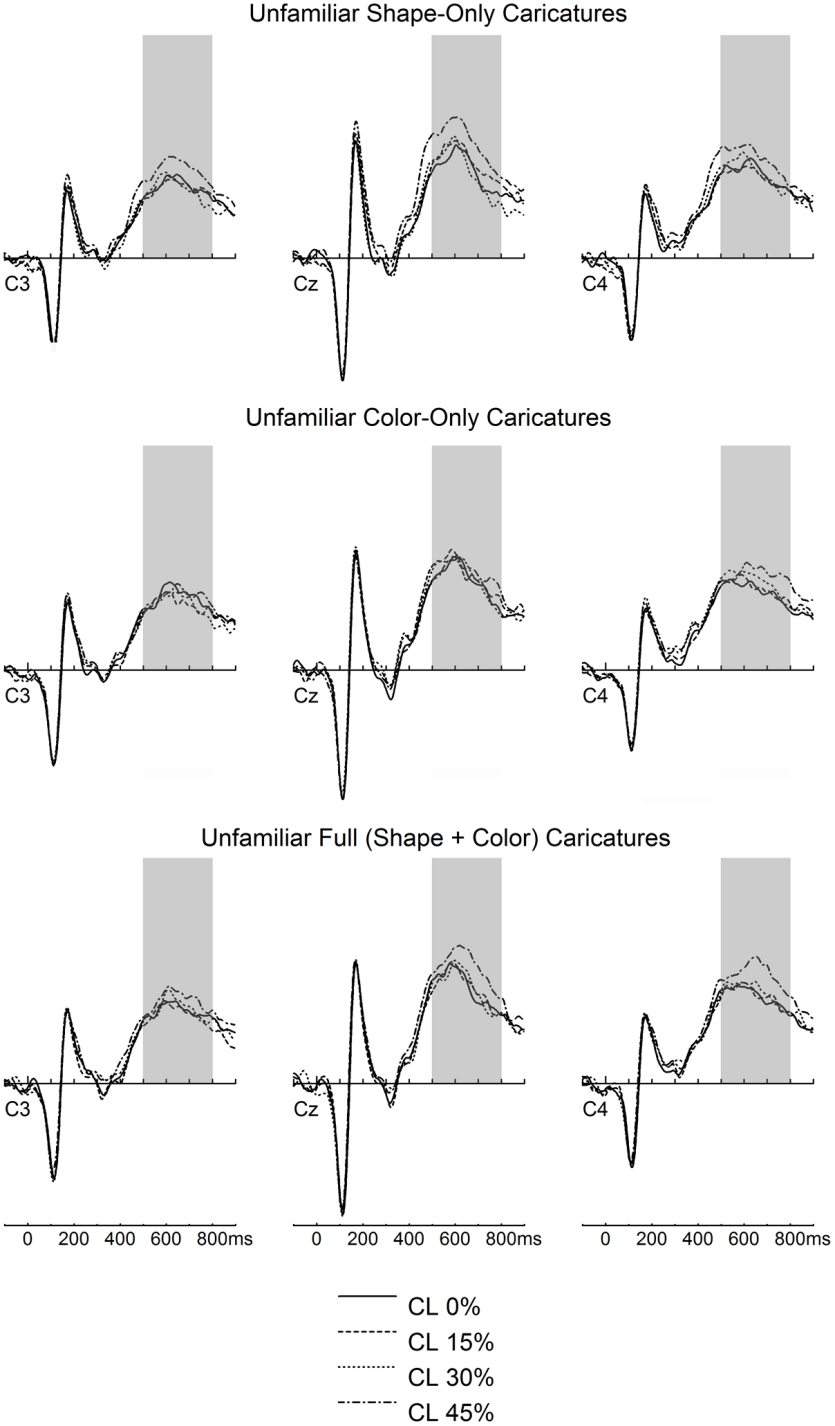
LPC caricature effects depicted for unfamiliar faces. The gray-shaded area depicts the time-window of interest (500–800 ms).

LPC amplitudes were larger for familiar compared to unfamiliar faces overall. Moreover, amplitudes were largest for larger levels of caricature level for faces containing caricaturing of shape (i.e. SC and FC; see Fig 5).

The ANOVA for LPC revealed a prominent main effect of familiarity, *F*(1,30) = 59.11, *p* < .001, η_p_^2^ = .663, due to stronger positivity for familiar compared to unfamiliar faces. Main effects of face type, *F*(2,60) = 3.43, *p* = .039, η_p_^2^ = .103, and caricature level, *F*(3,90) = 10.52, *p* < .001, η_p_^2^ = .260, were also found and interacted with one another, *F*(6,180) = 2.43, *p* = .028, η_p_^2^ = .075.

Separate analyses for each face type were performed to explore the interacted between face type and caricature level. Main effects of caricature level were found for shape, *F*(3,90) = 7.89, *p* < .001, η_p_^2^ = .208, and full (shape + color) caricatures, *F*(3,90) = 6.24, *p* = .001, η_p_^2^ = .172, due to linear trends (*F*[1,30] = 21.75, *p* < .001, η_p_^2^ = .420 for SC, and *F*[1,30] = 14.56, *p* = .001, η_p_^2^ = .327 for FC; see Fig 5). Note that for SC, the quadratic trend also reached significance, *F*(1,30) = 4.27, *p* = .047, η_p_^2^ = .125. There was no effect of caricature level for CC, *F*(3,90) = 0.52, *p* = .667, η_p_^2^ = .017.

## 4. Discussion

This is the first study to examine effects of selective caricaturing in either shape or color on recognition performance and neural correlates for pre-experimentally familiar and unfamiliar faces. Importantly, our use of pre-experimentally familiar faces allows inference about the recognition of real familiar (as opposed to experimentally familiarized) faces.

Despite earlier claims that caricaturing facilitates the recognition of known faces [15], we found no performance benefits of caricaturing for familiar faces. This finding is in line with more recent findings on pre-experimentally familiar shape caricatures [18], a result which in the current study extends to familiar faces caricatured in color. Lee and Perrett [19] argued that caricatures are advantageous for familiar face recognition when “processing is compromised in some way” (p. 749), e.g. when presentation time is very brief [17, 19]. In the current experiment, stimulus presentation duration was comparatively long, providing more time for participants to observe the stimuli. Moreover, to the extent that familiar (but not unfamiliar) face representations are robust against pictorial characteristics and manipulations (see e.g. [3, 48, 49]) small or absent advantages of caricaturing of pre-experimentally familiar faces can be expected.

In contrast, we found that performance for *unfamiliar* faces benefited from shape caricaturing. Fastest reaction times for unfamiliar shape caricatures complement previous reports (e.g. [31]), and the present tendency for highest accuracies for higher levels of unfamiliar shape caricatures (Table 1) is also broadly in line with previous findings [32]. Overall, the present behavioural findings support the conclusion that shape caricaturing facilitates identity-based processing of unfamiliar, but not familiar, faces.

In the following, we will first discuss ERP effects of caricaturing in some detail for each analyzed component before turning to ERP differences between familiar and unfamiliar faces. First, an unexpected finding was the slightly larger P100 for shape caricatures compared to the other face types (see Fig 2). The P100 is known to be highly sensitive to low-level pictorial characteristics, and to contrast in particular [50]. From that perspective, one might have expected—if anything—a slightly larger P100 for color caricatures (which have slightly increased contrasts; see Fig 2). We are currently unable to provide a convincing explanation for this small amplitude effect in the P100. It should be noted however that shape caricaturing did not elicit a P100 modulation in an earlier study [31]. In the absence of a replication of this effect, we therefore refrain from further speculation.

The present finding of slightly but systematically larger N170 for larger levels of caricaturing is in line with a previous finding [32], and was found here to be independent of the type of caricature. This finding could be interpreted in terms of enhanced structural encoding of caricatured faces, particularly when considering that the N170 has been specifically related to structural face encoding [21]. Note however that effects of caricaturing for the N170 were in previous studies smaller [32] and less consistent (e.g. [11, 24, 31]), particularly when compared to the large and consistent caricaturing effects in the subsequent P200 component.

Consistent with those previous findings here we found prominent effects of shape caricaturing for P200, which were even stronger for higher caricature levels. P200 has been associated with facial typicality (e.g. [51, 52]), especially in terms of norm or prototype deviation from a “face space” model [24, 53, 54]. Importantly, our finding of smaller P200 for shape caricatures was strongest for *unfamiliar* faces, complementing further findings on the importance of distinctive shape for identity-based processing of unfamiliar faces (e.g. [31]).

The present caricaturing effects on the N250 are also well in line with a number of previous findings. First, larger N250 for larger levels of shape caricaturing complements a previous finding [32], and extends it to faces containing caricaturing of color. The N250 is typically associated with the transient activation of stored mental face representations in memory and priming experiments [28, 55, 56] but has also been associated with the processing of particularly attractive, distinctive, or other-race faces [54, 57]. While the present effects of familiarity on the N250 (see below) replicate the usual finding of larger N250 amplitudes for familiar as compared to similar unfamiliar faces of the same category, it is important to keep in mind that different categories of faces can also affect ERPs in the N250 time-range.

Finally, LPC was larger for larger levels of caricaturing for faces containing shape caricaturing (i.e. SC and FC). Note that this is broadly in line with previous reports on larger LPC for caricatured stimuli [11, 24, 31, 32] and could reflect more efficient semantic processing of those faces compared to veridicals.

In terms of ERP effects for familiarity, the current findings of larger N250 and LPC for familiar compared to unfamiliar faces are in line with several previous reports (e.g. [11, 20, 24, 28, 34). Familiarity effects in terms of more negative earlier occipitotemporal components (N170 and P200) appear to be less consistent, but have also been occasionally reported for famous compared to unfamiliar faces. For instance, a larger N170 for famous compared to unfamiliar faces was found in the present study and another recent study [58]. Given the sensitivity of the N170 to physical stimulus attributes, an unambiguous interpretation of those effects as reflecting familiarity would require a balanced design with the same faces being familiar for one group of subjects but unfamiliar for another group of participants, and vice versa. Although this caveat may be too conservative, in view of the fact that both studies ensured equivalent luminance and contrast and used relatively large numbers of stimuli, it is important to consider when interpreting early ERP effects of familiarity.

Interestingly, we found also smaller P200 for familiar compared to unfamiliar faces. A recent study comparing effects of attractiveness on face learning found smaller P200 for attractive compared to unattractive faces [57]. The current finding of smaller P200 for familiar faces may thus be attributed to potential higher attractiveness of our familiar (i.e. here, famous) facial stimuli. Alternatively, the smaller P200 for familiar faces could reflect an early onset of the well-known N250 familiarity effect, also found in this study, which overlaps in time with the present P200. Further research is needed to refine this aspect.

One last point worth mentioning is that we did not find any supra-additive effects for caricaturing in shape and color. That is, effects of full (shape + color) caricaturing were never largest. This is potentially in contrast to a previous study [36]. However, it appears possible that those differences are related to either different procedures of stimulus manipulation or to the use of different EEG signals as dependent variables. Specifically, the stimuli in that study comprised morphs in which identity information had been changed by means of cross-identity morphing and not enhanced as is the case for caricatured stimuli in the current study. Moreover, Dzhelyova and Rossion’s [36] study involved an analysis of fast responses to periodic stimulation, whereas we analyzed ERPs to single presentations of faces.

In conclusion, our results complement findings on robust identification of highly familiar faces despite image manipulations (e.g. [3]). In contrast, and importantly, the current findings highlight the importance of idiosyncratic facial shape for identity-based processing of *unfamiliar* faces. This finding is particularly interesting for applied areas such as eye-witness testimony or occupational fields in which unfamiliar faces need to be identified (e.g. security- related professions such as passport controllers [2]). In line with this, McIntyre et al. [59] could show improved unfamiliar face matching performance for moderate levels of caricaturing (30%). Moreover, caricaturing may also be useful for potential training programs aimed at improving face recognition abilities for both persons in the normal population with poor face recognition skills (see e.g. [23]), and clinical patients with different varieties of face recognition impairments (see e.g. [60, 61, 62]). Recent work by Irons et al. [63] could show promising results for similar applications. Moreover, the current finding of earlier ERP modulation by shape than color caricaturing complements previous reports [29, 31]. Overall, the current findings indicate robust recognition for pre-experimentally familiar faces and highlight the importance of distinctive shape for identity-based processing of unfamiliar faces.

## Supporting Information

S1 Dataset Spreadsheets including all datasets for behavioral and EEG results. (XLSX)

S1 Text Supplementary text file with descriptions for data organization within S1 Dataset. (TXT)
